# Waste-derived single-cell protein in Nigeria: current status, translational gaps, and development opportunities

**DOI:** 10.1186/s12934-026-03081-z

**Published:** 2026-08-15

**Authors:** Daniel Clement Agurokpon, Ntui Okam Njok, Yves kily Hervé Fagnidi, Nneka Ndifon Agbiji

**Affiliations:** 1 Department of Microbiology, University of Cross River State, Calabar, Nigeria; 2 Department of Science Laboratory Technology, University of Calabar, Calabar, Nigeria; 3 Science and Technology Training and Research Unit, Alassane Ouattara University of Bouaké, Bouaké, Ivory Coast

**Keywords:** Single cell protein, Waste valorisation, Agro-industrial waste, Microbial protein, Feed-grade protein, Circular bioeconomy, Sub-Saharan Africa

## Abstract

This review examines the current state of single-cell protein (SCP) research based on waste-derived substrates in Nigeria, with emphasis on substrate choice, microbial systems, bioprocess strategies, performance reporting, and prospects for commercial translation. Evidence from Nigeria-focused studies shows that a broad range of locally available waste streams, particularly those containing cassava peels, yam peels, cereal byproducts, fruit wastes, and sawdust, can support microbial growth and protein content improvement when yeasts, filamentous fungi, bacteria, and, to a lesser extent, mixed cultures are used. Most studies clearly demonstrate biological feasibility, and several also report reductions in the levels of fibre, cyanide, or other antinutritional factors. A relatively small number of studies extend to short-term feeding trials, providing early support for feed-oriented application. Despite this promise, the Nigerian literature remains dominated by laboratory-scale studies based largely on simple solid-state or batch submerged fermentation systems. Important translational elements, including strain robustness, process standardisation, downstream recovery, local techno-economic assessment, and regulatory preparation, remain poorly developed. Inconsistently reported protein-related outcomes have been reported, with many studies relying on crude or soluble protein measurements without a deeper assessment of amino acid quality, digestibility, or product safety. As a result, a clear gap persists between demonstrated laboratory feasibility and market-ready SCP production.

## Introduction

Single-cell protein (SCP) production is gaining widespread recognition as a sustainable response to the combined challenges of increasing global protein demand and organic waste accumulation. It refers to the microbial biomass, or protein derived from microbial biomass, obtained from microorganisms such as bacteria, yeasts, fungi, and microalgae cultivated for use as food or feed [1]. SCP is distinct from fermented or protein-enriched waste substrates, in which microbial activity increases the protein content of the original material without clear recovery of microbial biomass as the principal product. Existing global reviews show that SCP research involves diverse microorganisms, feedstocks, and process configurations for microbial protein production, improved environmental health and the promotion of the bioeconomy [2, 3]. While SCP systems in regions such as Europe, North America and Asia are highly discussed in relation to pilot-scale development, defined regulatory pathways, and commercial feed or food applications, progress is unclear in most countries, including Nigeria. It is also not well established whether SCP production research is still dominated by laboratory examinations such as substrate screening, protein enrichment, and early proof-of-concept.

Nigeria and other sub-Saharan African countries generate large quantities of organic waste and underutilised biomass from major crops such as cassava, yam, rice, maize, and fruits. Recent agricultural data indicate that Nigeria produced 21.90 million tonnes of cassava, 18.70 million tonnes of yam, 17.35 million tonnes of maize, and 13.04 million tonnes of rice [4]. These figures indicate a substantial biomass base within the agricultural system. In addition to production residues, significant amounts of food waste are generated at the household level. The UNEP Food Waste Index Report (2021) estimates that Nigeria produces approximately 37.9 million tonnes of household food waste annually. The report also indicates a per capita waste generation of 189 kg per person per year, placing the country among the highest globally [5]. These data show the scale of underutilised biomass in Nigeria. Improper disposal of these wastes contributes to environmental challenges, including soil and water pollution, greenhouse gas emissions, and public health risks. Moreover, these materials contain carbohydrates and other nutrients that support microbial growth. Their conversion into SCP provides a pathway for waste valorisation and may contribute to addressing protein deficiency and feed shortages.

The commercial success recorded in countries such as the United Kingdom (e.g., Quorn), the United States (e.g., Calysta’s FeedKind), and Finland (e.g., Solar Foods’ Solein) demonstrates that microbial protein can achieve market viability [6–9], illustrating a proven pathway from fermentation technology to commercial feed and food ingredients. These precedents are particularly relevant for countries that generate large amounts of organic waste and face persistent protein supply challenges. However, despite the abundance of such resources, many countries are yet to capitalise on this significant opportunity. Nigeria, for example, continues to rely heavily on imported high-protein feed inputs such as fishmeal and some soybean-derived products [10]. This leaves a large amount of waste to remain underutilised, sometimes untapped, and poorly managed in most rural settings, contributing to environmental burden and economic crises. Trade data show that Nigeria imported approximately 9258.9 tonnes of fishmeal-related products in 2023, which is valued at approximately US$20.05 million, while imports of soybean oil cakes and other soybean residues reach approximately 2956.4 tonnes, which is valued at US$3.11 million [11, 12]. This uncontrolled dependence exposes the country to global price volatility, places pressure on foreign exchange reserves, and limits progress toward food self-sufficiency. SCP could reduce pressure on imported protein inputs only if locally produced microbial biomass can achieve competitive cost, adequate safety, acceptable amino acid quality, and a reliable supply at the feed industry scale.

Although several reviews have examined SCP from global perspectives, most of them focus on broad technological trends, substrate classes, fermentation strategies, sustainability, or techno-economic prospects [3, 7, 13–15] rather than on Nigeria or sub-Saharan Africa as distinct translational settings. For example, Onyeaka et al. [1]. mapped overall SCP research trends, Bajić et al. [13]. reviewed microbial protein production from agro-industrial residues and byproducts, and Sekoai et al. [3]. examined food waste valorisations into SCP, with only a brief reference to the African context. A partial exception is Yafetto et al. [16], who reviewed agro-industrial animal feed through solid-state fermentation globally and in a Ghanaian case study. However, their focus is on upgrading the feed value of the fermented substrate itself and reducing anti-nutritional factors and raising crude protein in situ rather than on single-cell protein as a distinct, recoverable biomass product, and their scope is limited to solid-state systems in a different national context. These studies provide a broad understanding of SCP production at the global level. However, they do not critically assess Nigeria-specific waste streams, distinguish true microbial biomass production from fermentation-based protein enrichment in the local literature, or evaluate whether Nigerian studies are approaching the level of process maturity seen elsewhere. Hence, a Nigerian-focused appraisal of waste-derived SCP research is presented here, with attention given to substrate suitability, microbial systems, bioprocess strategies, and the maturity of the evidence for practical development.

## Review strategy

This study employed a narrative review methodology with a descriptive analytical approach to synthesise the literature on waste-derived SCP in Nigeria. The narrative design was selected to allow a flexible yet structured integration of findings across studies that differ in substrate type, microbial systems, fermentation strategies, and outcome measures. Given the heterogeneity of the Nigerian SCP literature, including differences in experimental design, reporting metrics, and application focus, a narrative synthesis was considered more appropriate than a highly restrictive systematic framework. The aim was therefore to map the Nigerian evidence base, identify patterns in substrate use and microbial performance, and evaluate the extent to which current studies support translational development. The literature search was conducted using the Scopus database and Google Scholar. Scopus was used as the primary indexed database because of its broad coverage of peer-reviewed scientific literature, while Google Scholar was used to capture additional records, including regionally published studies and materials that may not be fully indexed in highly controlled databases.

Search terms were initially developed around core concepts and combined using appropriate Boolean operators and wildcards. These included “single-cell protein,” “microbial protein,” microbial biomass,” “wastes,” “agro-industrial residues,” “fermentation,” and “Nigeria.” As the search progressed, the strategy was iteratively refined to improve coverage of the Nigerian context. This involved incorporating more specific substrate-related terms such as “cassava,” “yam,” “fruit,” “pineapple,” “orange,” “mango,” “plantain,” “rice,” “potato,” and “sawdust,” as well as microorganism-related terms, including broad groups such as ‘yeast,’ ‘bacteria,’ and ‘fungi,’ alongside representative genera and species (e.g., *Saccharomyces*) identified during the search process. These additions were informed by patterns observed in the retrieved studies and were used to capture relevant literature that may not have been indexed under broader SCP-related keywords. In addition, reference lists from relevant articles were manually screened to identify further studies not captured through database searches. No fixed publication timeframe was applied. This decision was made to capture the full scope of available Nigerian research, given that the field remains relatively limited and unevenly distributed over time. Applying strict temporal restrictions could exclude foundational or contextually important studies relevant to understanding the development trajectory of SCP research in the country. The final search was conducted on the 6th of November, 2025.

Study selection followed a manual relevance-based screening process. Retrieved records were first assessed on the basis of title and abstract, after which full-text evaluation was conducted for studies considered potentially relevant. The inclusion criteria were defined to ensure alignment with the objectives of the review. Studies were included if they reported experimental investigations of SCP production, microbial growth, or protein enrichment using waste-derived substrates within the Nigerian context. This included laboratory-scale fermentation studies, as well as selected feeding trials in which SCP-related materials were evaluated for practical application. In addition, relevant review articles and international case studies were included where necessary to provide global context and comparative insight. Studies were excluded if they did not relate to SCP or microbial protein production, if they focused entirely on non-Nigerian systems without comparative relevance, or if they were duplicate records. Only articles published in English were considered.

Following study selection, relevant data were extracted and organised according to key analytical variables. These included substrate type, microorganisms used, fermentation method, reported outcomes (such as biomass yield, crude protein, soluble protein, or related indicators), and the nature of the evidence (e.g., direct biomass production, protein enrichment, or growth-support data). The extracted information was then examined using a descriptive analytical approach, with emphasis on identifying patterns in substrate suitability, microbial utilisation, process strategies, and the level of evidence supporting practical application. Given the narrative nature of the review, certain limitations must be acknowledged. The reliance on manual screening and relevance-based inclusion introduces a degree of subjectivity and potential selection bias. In addition, the absence of a formal systematic protocol means that some relevant studies may not have been captured. However, the use of multiple search platforms, iterative keyword refinement, and reference tracking was intended to reduce this risk and ensure a sufficiently comprehensive representation of the Nigerian SCP evidence base.

## Overview of major waste streams relevant to SCP production

### Concept of substrate suitability for SCP production

**Fig. 1:**
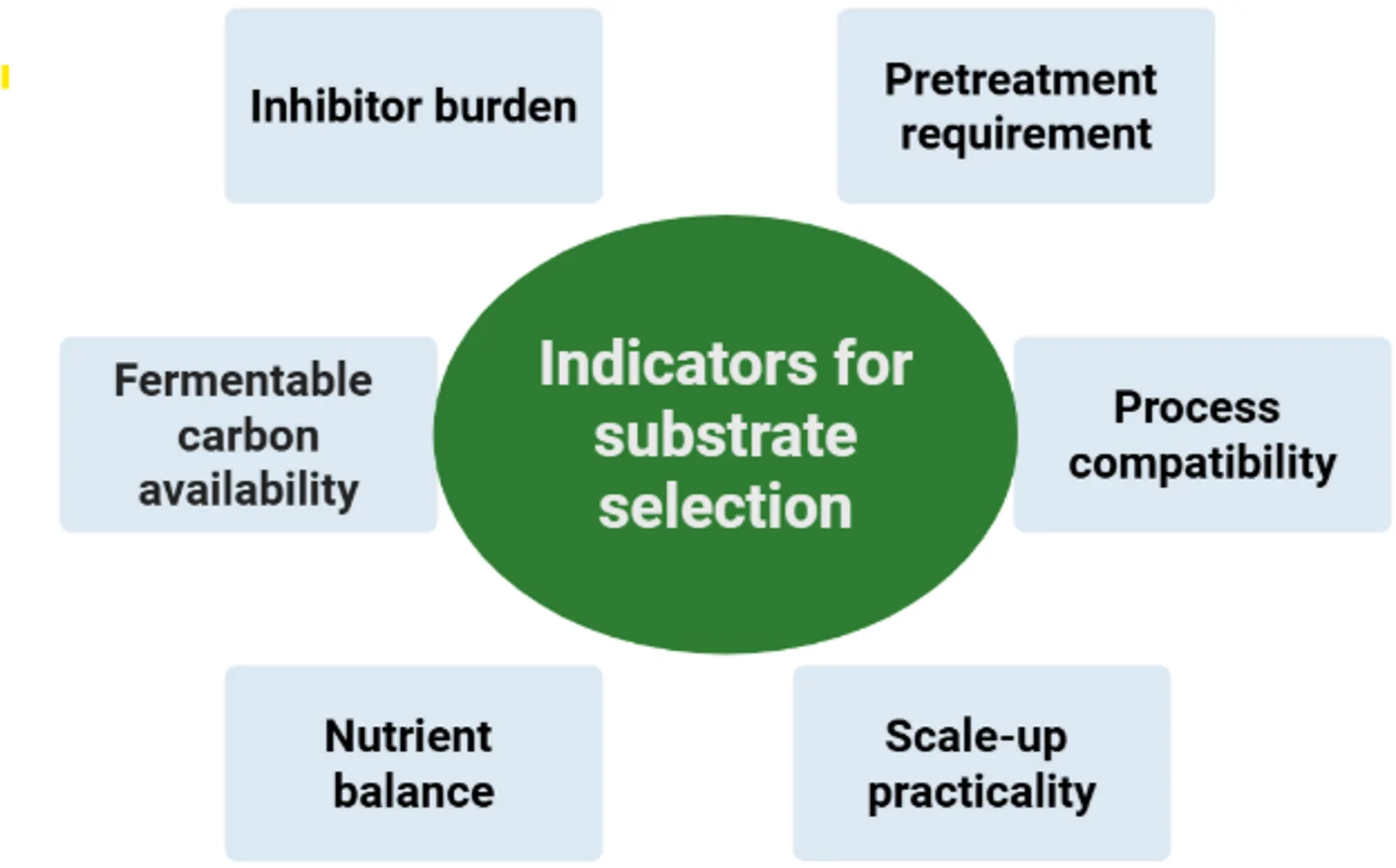
Conceptual framework used in this review to interpret substrate suitability for SCP production

In the context of SCP production, substrate suitability refers to the extent to which a waste material can support efficient, safe, and economically realistic microbial biomass formation. A suitable substrate should be available in sufficient quantity and provide carbon in a form that is accessible to selected microorganisms, either directly or after feasible pretreatment [17]. In other words, substrate suitability is shaped by several interrelated factors, including fermentable carbon availability, nutrient balance, moisture content, physical structure, inhibitor burden, pretreatment requirements, and compatibility with the intended fermentation mode. Substrates rich in simple sugars or readily hydrolysable carbohydrates are generally more favourable because they can be assimilated more easily by yeasts, fungi, or bacteria [18], whereas highly lignocellulosic materials often require more intensive hydrolysis before efficient SCP production can occur [19]. Likewise, substrates containing inhibitory compounds, such as toxic metabolites or antinutritional factors, may require detoxification before microbial growth becomes reliable [20]. From this perspective, a substrate may therefore be biologically usable but still not highly suitable for near-term SCP development if it demands costly pretreatment, unstable supply, or complex downstream handling. In line with this concept, substrate suitability is determined by several parameters, such as fermentable carbon accessibility, nutrient balance, inhibitor burden, pretreatment requirements, process compatibility, and scale-up practicality (see Fig. 1), in addition to waste availability. Hence, the substrates relevant to SCP production may be grouped according to their dominant biochemical characteristics and degree of microbial accessibility, since these properties strongly influence fermentability, pretreatment burden, and scale-up promise.

### Grouping of Nigerian waste streams by biochemical composition and microbial usability

**Fig. 2:**
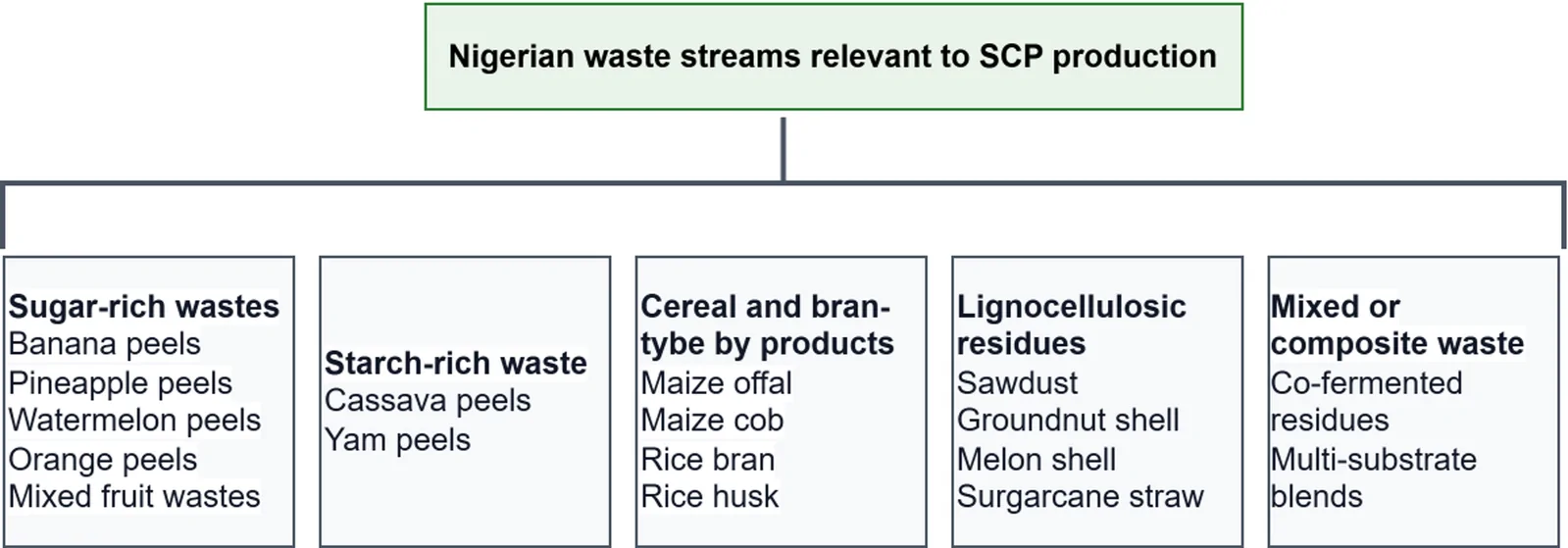
Classification of Nigerian waste streams relevant to SCP production

As previously established, waste streams relevant to SCP production can be grouped more meaningfully by their biochemical composition and microbial usability. In this sense, the origin of crops alone does not affect their classification. This is because wastes derived from different crops may behave similarly during fermentation if they share comparable biochemical characteristics [21], as established in Sect.  3.1. Grouping substrates in this way allows a more functional comparison rather than simply listing them by source. In the Nigerian context, waste streams relevant to SCP production can be grouped as sugar-rich wastes, starch-rich wastes, cereal and bran-type byproducts, lignocellulosic or highly fibrous residues, and mixed or composite wastes (see Fig. 2).

Sugar-rich wastes may include many fruit-processing residues and kitchen-derived peels, such as banana, plantain, orange, pineapple, and similar fruit wastes. These substrates are generally favourable for SCP production because they contain readily fermentable sugars that can often be assimilated directly by yeasts and other microorganisms with little or no severe pretreatment [22]. However, these wastes are also highly perishable, compositionally variable, and often seasonally available, which can limit storage, logistics, and supply stability. Even though they are among the most biologically favourable substrate groups, their scale-up value depends strongly on preservation, aggregation, and handling systems, which may be a concern. To achieve the optimum yield, timely processing is needed.

Starch-rich wastes may include cassava-derived residues, yam-derived residues, and other carbohydrate-dense root and tuber byproducts. These are among the most important Nigerian substrate groups because they are deeply embedded in the country’s food system and generate large volumes of peels and processing waste. Additionally, they are attractive for SCP because of their substantial fermentable carbon potential [13], but hydrolysis, detoxification, or nutrient supplementation is often needed before efficient microbial utilisation becomes possible [23]. Despite this, their high carbohydrate content [24] and broad local availability make them strong candidates for near-term development.

Cereal and bran-type byproducts may include rice milling byproducts, maize offal, maize cob, cereal brans, sorghum fermentation residues, and related grain-processing materials. This group occupies an intermediate position between readily utilizable sugar- or starch-rich wastes and more recalcitrant lignocellulosic materials [25]. Bran-like residues and some grain-processing byproducts often retain a more favourable mixture of carbohydrates, fibre, and residual nutrients than husks, shells, or woody wastes do. This makes them more attractive for fermentation than raw structural biomass. Their value depends not only on the nutrient profile but also on the fact that many are generated in semi-industrial or organised processing settings, which can improve collection, standardisation, and supply consistency relative to widely dispersed household waste. Their microbial usability is therefore often moderate to high, depending on the extent of the fibre and the need for size reduction or mild pretreatment.

Lignocellulosic or highly fibrous residues may include husks, straw-like residues, sawdust, shells, bagasse, woody wastes, and related materials in which cellulose and hemicellulose are embedded within a lignin-rich matrix. These substrates are abundant and potentially valuable, but they are generally the least directly utilizable for SCP because microbial access to fermentable sugars is constrained by structural recalcitrance [26]. In most cases, physical, chemical, enzymatic, or combined pretreatment is required before efficient fermentation becomes possible. Residues such as sawdust, rice husk, groundnut husk, and similar fibrous wastes may therefore be highly interesting but less attractive for immediate SCP deployment under low-technology or decentralised conditions. Their strongest relevance may lie in more advanced biorefinery approaches or in long-term process development rather than near-term commercial SCP production.

Mixed or composite wastes are also important because co-substrate systems have been shown to improve nutrient balance, dilute inhibitors, and stabilise fermentation performance [27, 28]. This is also important in Nigeria because many residues occur together spatially, seasonally, or within the same household and processing environments. In such cases, mixed-substrate systems may perform better than single-substrate systems do, especially when one waste is carbon rich but nitrogen poor or when blending reduces spoilage losses and improves substrate consistency [29, 30]. From a suitability perspective, compared with isolated feedstocks, mixed wastes may sometimes offer a more practical route for SCP production, although their performance depends on how well the combined substrates complement one another chemically and microbiologically.

### Comparative ranking of Nigerian substrates for near-term SCP development

Figure 2 is used as the basis for this ranking, while an example study is presented in Table 1. Among the sugar-rich substrates, fruit waste stands out for a specific, actionable reason, as two independent studies show that simple process choices (a nitrogen source, or the choice of fruit) move protein output substantially, even though the two studies quantify that output differently and cannot be pooled into one figure. Oshoma and Eguakun-Owie [31] grew *Aspergillus niger* on banana waste and measured gravimetric dry-weight biomass alongside Kjeldahl-based protein content, reporting biomass = 2.29 ± 0.02 g/L and protein = 0.57 ± 0.01 g/L without supplementation, rising to biomass = 3.20 ± 0.02 g/L and protein = 0.79 ± 0.04 g/L with ammonium nitrate. Abarshi et al. [32] used submerged fermentation with *Saccharomyces cerevisiae* and Kjeldahl-based SCP quantification to show that pineapple peel hydrolysate outperformed watermelon peel hydrolysate (9.8% vs. 2.42% SCP). The practical takeaway is less about ranking these two studies against each other and more about what they jointly demonstrate: fruit-waste protein yield responds predictably to cheap process changes, a promising sign for low-cost scale-up rather than an obstacle requiring further method-matching before it can be acted on.

Among starch-rich substrates, yam peels carry the strongest Nigerian evidence, for two distinct reasons that should not be merged into one. Aruna et al. [33] used Kjeldahl-based crude protein and a non-protein-nitrogen-corrected true-protein measure (total nitrogen by Kjeldahl digestion, with non-protein nitrogen removed by trichloroacetic acid (TCA) precipitation and subtracted before applying the nitrogen-to-protein conversion factor) to show that fermenting yam peels with *S. cerevisiae*, with ammonium sulfate present, raised crude protein from 6.60% to 15.54%, true protein from 4.38% to 13.37%, and improved the amino acid profile which directly point to protein enrichment. Abdurrazaq et al. [34], by contrast, reported only a growth comparison. Yam peel supported denser *A. niger* growth (OD_650_, 0.40) than yeast extract peptone dextrose (OD_650_, 0.23). This points to yam peel as a promising low-cost growth substrate, though the comparison should be read with some caution, since a crude yam-peel extract can itself contribute background turbidity to an OD reading, and it is not clear from the source whether an uninoculated yam-peel blank was used to correct for this. It is not, in any case, evidence that the substrate enriches protein. The two studies make a stronger case for yam peels than either would alone. One shows the protein-enrichment ceiling is high, the other shows the raw substrate supports fungal growth at least as well as an expensive commercial medium, both pointing to yam peels as a substrate worth prioritising for a combined growth-and-enrichment pilot.

Cassava-derived substrates matter more for their strategic importance than for uniformly strong performance. Their importance comes from their abundance and repeated appearance in Nigerian fermentation studies, but the evidence shows that they are often condition-dependent rather than automatically high-performing. The two findings below are measuring different things, which is part of why they seem to disagree. Anigboro et al. [35] first subjected cassava peels to solid-state fermentation with *Rhizopus oligosporus*, then used the resulting fermented cassava-peel extract as a nutrient source to grow *S. cerevisiae*. Compared with the unfermented control (OD_600_, 0.113 ± 0.001), *S. cerevisiae* grown on *Rhizopus oligosporus*-fermented cassava-peel extract reached OD_600_ 0.465 ± 0.005, indicating that fermentation made the peel a substantially better growth substrate. Separately, in the *Pleurotus sajor-caju* mycomeat study, cassava peel was one of the poorest-performing substrates by dry-weight biomass yield, reaching only 0.02 g/L at 7 days and 0.05 g/L at 14 days for the mutant strain, well below the other substrates tested [36]. In other words, cassava peel can support strong microbial growth in one system while yielding poor biomass in another which is a fungus- and process-specific result, not a general verdict on the substrate. This is consistent with the known limitations of cassava residues, including cyanogenic compounds and low nitrogen balance [37, 38]. Related cassava-root fermentation studies have also shown that fermentation can markedly improve protein quality while reducing cyanide, but again, this points to the need for conditioning and detoxification rather than effortless direct use. For that reason, cassava residues are best treated as high-priority but process-sensitive substrates rather than uniformly top-performing ones.

Within cereal and bran-type byproducts, the evidence supports an intermediate but promising position, and includes one of the few actual feeding-trial results in this literature. Anigboro et al. [39] used a colorimetric soluble-protein assay to show that solid-state fermentation of maize offal with *R. oligosporus* substantially increased soluble protein relative to a control (203.87 ± 13.30 mg/g), reaching 421.73 ± 3.00 mg/g at pH 3. Ndego et al. [40] used a biuret assay to show that fermenting maize cobs with *R. oligosporus* increased soluble protein from approximately 7.6 mg/g (unfermented control) to approximately 10.9 mg/g at 10% inoculum. And, notably, went a step further than most studies (see Table 1) by feeding the fermented product to broilers, reporting that chicks on the 10%-inoculum fermented-cob diet and the control both gained 1.4 ± 0.1 kg over the trial. Both studies report biochemical enrichment rather than direct SCP biomass yield, but Ndego et al.’s animal-performance data is exactly the kind of downstream validation most of the reviewed literature still lacks, which makes maize-based byproducts a stronger near-term candidate than raw fibrous wastes for feed-oriented microbial upgrading.

The strongest direct evidence of biomass yield, measured gravimetrically, as dry weight, rather than as a protein percentage among agro-industrial substrates comes from mycomeat studies using *Pleurotus sajor-caju*. Adetunji and Adejumo [36] compared a UV-derived mutant strain against the wild type across several substrates in solid-state fermentation, and found that palm kernel meal supported the highest mycomeat biomass production, reaching 10.50 g/L at 7 days and 17.20 g/L at 14 days in the mutant strain, while rice bran also performed well (6.20 g/L and 10.40 g/L, respectively). Groundnut shells also supported relatively high biomass, whereas cassava peel performed poorly. These results suggest that not all structurally complex wastes are equally weak; some nutrient-rich byproducts, especially those with more balanced carbon and nutrient profiles, may outperform more abundant but inhibitor-burdened starch wastes in certain fungal systems.

On the other hand, highly lignocellulosic substrates such as rice husk, sawdust, shells, and similar fibrous wastes have lower potential for immediate SCP deployment. Their major limitation is not abundance but structural recalcitrance. Even when fermentation improves their biochemical profile, the reported outcomes usually reflect the upgrading of a difficult substrate rather than straightforward SCP production. For example, the results of the maize cob and cassava-root studies above show that fermentation can increase soluble protein and feed value, but such substrates still require more process intervention than sugar-rich fruit waste or well-balanced byproducts [18]. The same logic applies even more strongly to sawdust and rigid shells, which generally require stronger hydrolysis or pretreatment before microbial access to fermentable carbon becomes efficient [41]. Thus, these residues remain scientifically interesting and potentially valuable in the long term, but they are weaker near-term candidates for low-technology or decentralised SCP deployment in Nigeria.

In all, yam peels, selected fruit wastes, maize offal, and nutrient-rich byproducts such as palm kernel meal and rice bran appear most suitable for near-term development, whereas cassava residues remain important but are process sensitive, and highly lignocellulosic materials are better suited to longer-term or advanced biorefinery approaches. Therefore, substrate selection should be guided by the framework outlined in Sect.  3.1.

## Evidence from key findings and case studies

### Microbes explored for SCP production from Nigerian wastes

The Nigerian literature on waste-derived SCP is dominated by yeasts and filamentous fungi, while bacterial systems and mixed cultures remain relatively limited. This distribution is important because it shapes both the kinds of substrates that have been successfully explored and the type of evidence available for judging scale-up promise. In the reviewed studies, yeasts and fungi account for most of the direct biomass, protein enrichment, and biochemical-upgrading results, whereas bacterial and mixed-culture systems appear in fewer studies and are less well characterised. Recent SCP reviews also note that different microbial groups have distinct substrate preferences and process implications, making the organism class a useful basis for comparison rather than a simple study-by-study list [31]. Yeast-based systems in the Nigerian context are dominated by *S. cerevisiae*, in addition to mixed yeast cultures such as those derived from palm wine. In these studies, yeast fermentation is frequently associated with improved protein content, enhanced amino acid profiles, and increased soluble protein in waste substrates, including yam peels, cassava peels, fruit wastes, and rice husk (Table 1). In several cases, prior substrate conditioning or cofermentation further improved yeast growth performance, indicating that carbon accessibility and a reduction in inhibitory compounds are key determinants of process efficiency. However, much of the available evidence is based on protein enrichment, increases in soluble protein, or growth-support indicators rather than on consistently reported direct biomass yields.

Filamentous fungi are the most versatile group in the current Nigerian evidence base. They are reported across a wide range of substrates, including sugar-rich wastes, starch-based residues, cereal byproducts, and lignocellulosic materials, as seen in the mycomeat production systems (Table 1). This broad substrate coverage suggests greater flexibility than yeast-based systems. Across the reviewed studies, genera such as *Aspergillus*, *Rhizopus*, *Trichoderma*, and *Pleurotus* are consistently associated with biomass formation, protein enrichment, and substrate upgrading. Fungal systems also provide the clearest evidence of direct biomass production, particularly in mycomeat-oriented applications, where substantial yields have been reported on selected agricultural substrates (Table 1). Moreover, performance varies considerably with substrate type, with some materials supporting strong growth and others remaining poorly utilised. Despite this versatility, much of the fungal evidence remains at the level of laboratory-scale enrichment or feed-oriented applications. In addition, filamentous systems introduce process considerations such as contamination control and, in some cases, the need to manage potential mycotoxin risks under suboptimal conditions. These factors are relevant when scale-up is considered beyond experimental settings.

Bacterial systems remain underexplored. The main Nigerian example identified here is the study of *Bacillus subtilis* on melon, groundnut, and walnut shell wastes, which revealed the highest cell biomass on groundnut shells and generally revealed that agro-shell wastes could serve as substrates for bacterial SCP production [42]. This study [42] is useful because it broadens the organism base beyond that of yeast and fungi, but it also highlights the thinness of the bacterial evidence. At present, bacteria cannot be judged as strongly as fungi or yeasts in the Nigerian context because the evidence base is too narrow and mostly limited to optimisation on a small set of shell wastes.

Mixed cultures occupy an interesting but methodologically weaker position. Their main potential advantage is functional complementarity. Compared with single organisms, mixed systems can broaden substrate use, improve nutrient capture, reduce inhibitory effects, and sometimes result in greater protein gains. The results of a 2025 rice-husk study provide a good example. Co-fermentation with baker’s yeast, palm wine yeast, and *R. oligosporus* increased the soluble protein concentration from 4.7 mg/g in raw husk to a much higher level under cofermentation, with the authors reporting a 183% increase over that in untreated husk [43]. Broader SCP-related literature also supports mixed-culture advantages for fruit and peel wastes, where complementary metabolism can improve overall bioconversion. However, these advantages come with trade-offs. Compared with monocultures, mixed cultures are more difficult to standardise, more difficult to reproduce across batches, and less easy to interpret mechanistically [44–46]. This makes them appealing for exploratory waste valorisation but more challenging for rigorous scale-up unless community composition and process stability are better controlled.

### Bioprocessing strategies and operational conditions

At present, Nigerian SCP-related studies rely heavily on laboratory-scale batch fermentation, with solid-state fermentation (SSF) and submerged fermentation (SmF) identified as the two process modes dominating the literature. The choice between them is not trivial because each mode favours different substrate types, operational conditions, and downstream possibilities. However, the reviewed evidence shows that SSF is preferred when the substrates are starchy, semisolid, or fibrous, whereas SmF is used mainly when wastes are hydrolysed, are sugar-rich, or are more easily dispersed in liquid culture. SSF is the dominant strategy, especially for yam peels, cassava peels, maize offal, maize cobs, rice bran, rice husk, and mycomeat systems based on *Pleurotus* species (see Table 1). This mode is compatible with low-moisture agroresidues, uses relatively simple equipment, and often aligns well with the handling characteristics of peels, brans, cobs, and fibrous substrates. SmF is less common but operationally more controlled. It appears mainly in studies using fruit wastes, hydrolysed peel extracts, and shell-based bacterial systems. In a pineapple and watermelon peel study, *S. cerevisiae* was cultivated in liquid fruit hydrolysates under defined nutrient supplementation and pH adjustment, and the main outcomes were reported as broth crude protein and SCP percentage [32]. Similarly, in a shell-waste study with *Bacillus subtilis*, a batch fermenter with controlled aeration (2 vvp), agitation (300 rpm), and pH adjustment to 7.0 was used, in addition to optimisation of the inoculum size, fermentation period, carbon supplementation, and nitrogen source [42]. These examples show that SmF allows clearer manipulation of growth conditions and easier measurement of broth-based responses, particularly when the substrate is already soluble or hydrolysed. The trade-off is that compared with SSF, SmF usually demands more water, stricter sterility, more agitation and aeration, and better reactor control. This likely explains why SmF appears mainly in studies using fruit hydrolysates, treated cassava peel extracts, or shell-based liquid systems rather than in the wider body of the peel, bran, and fibrous residue (see Table 1).

### Strain robustness, contamination risk, inhibitor tolerance, and limits of scalability

A major limitation of the current Nigerian SCP-related literature is that it demonstrates substrate feasibility more often than process robustness does. Most of the reviewed studies show that microorganisms can grow on selected wastes or improve their protein-related characteristics under controlled laboratory conditions, but researchers rarely test whether those organisms would remain reliable under more variable operational conditions associated with scale-up. For example, the fruit-peel study by Abarshi et al. [32]. used submerged batch fermentation of hydrolysed pineapple and watermelon peels under defined pH adjustment and nutrient supplementation, and the shell-waste study by Omogbai and Obazenu [42] optimised the inoculum size, incubation time, pH, carbon source, and nitrogen source for *Bacillus subtilis*. Similarly, the maize offal, maize cob, cassava-peel, and rice-bran studies adjusted for selected variables such as pH, fermentation duration, moisture, or supplementation but retained laboratory-scale batch systems rather than pilot or repeated-production studies [35, 39, 40, 47]. This means that the Nigerian evidence base currently says much more about whether local wastes can support microbial activity than about whether the selected strains are sufficiently robust for reproducible industrial operation.

One aspect that remains poorly addressed is strain robustness under fluctuating process conditions. Several studies optimised growth under a narrow range of selected laboratory conditions, but few have investigated how performance would change if the substrate quality varied across batches or if the operating environment became less controlled. For instance, Abarshi et al. [32]. adjusted fruit hydrolysate fermentation to a pH of 5.5, inoculated it with 3 × 10^8^ cells/mL, and incubated it for 20 days, whereas Omogbai and Obazenu [42] reported that the yield of bacterial biomass depended strongly on the pH, inoculum size, incubation time, and nitrogen source, with the best performance occurring at pH 7.0 and 72 h. These results show signs of parameter sensitivity, but they do not establish whether the strains would remain stable under operational disturbance, repeated runs, or more heterogeneous industrial feedstocks. Similarly, Adekunle et al. [47]. optimised crude protein improvement in rice bran by adjusting the temperature and fermentation duration but did not report interbatch consistency or robustness under variable substrate compositions. Thus, the reviewed studies are informative for feasibility but still have limited reproducibility.

A second important issue is contamination risk, which is relevant in fermentation systems based on agro-residues, peels, and heterogeneous household or processing wastes. Many of the studies used either solid-state or small submerged batch systems after sterilisation or autoclaving, but they did not report contamination frequency, sterility failures, interbatch microbial competition, or recovery from contamination events. A few typical studies involve a fruit-peel submerged system [32], shell-waste bacterial fermentation [42], and *Pleurotus* mycomeat systems [36, 48], all of which rely on controlled laboratory preparation and incubation. The absence of contamination reporting does not imply that contamination was absent; rather, it means that contamination resistance has not yet been treated as a core performance parameter. This is a significant gap because industrial SCP production requires strains and processes that remain reliable when sterility is more difficult to maintain than in small laboratory systems, as discussed elsewhere [49].

These studies provide only limited direct evidence on inhibitor tolerance, even though the inhibitor burden is highly relevant to the local substrate base. Cassava-derived systems illustrate this clearly. As summarised in Table 1, yeast growth substantially increased when cassava peels were subjected to prior fungal treatment, indicating that preconditioning enhances substrate suitability. Similarly, the fermentation-based upgrading of cassava residues is associated with increased protein-related metrics and reduced cyanide levels, reinforcing the need for detoxification before efficient microbial utilisation [35]. Evidence from fungal systems points in the same direction. Mycomeat studies using *Pleurotus sajor-caju* report very low biomass yields on cassava peel relative to those on other substrates, with the limitation attributed to the presence of hydrocyanic acid [48]. These findings indicate that inhibitor management is a central constraint in cassava-based SCP systems. However, the current literature does not clearly distinguish between true microbial tolerance to inhibitory compounds and improved performance following substrate detoxification, leaving an important gap in the understanding of this process.

Another major gap is the limited use of strain improvement and advanced strain-selection strategies. Most studies rely on naturally available or conventionally maintained strains of *S. cerevisiae*, *R. oligosporus*, *(A) niger*, *Pleurotus* spp., or *(B) subtilis* [32, 33, 35, 39, 42]. Notably, Adejumo et al. [48]. reported that improved UV strain enhanced the biomass production of *Pleurotus sajor-caju* on several agricultural substrates, including palm kernel meal, rice bran, and groundnut shell. This is important evidence that strain improvement can materially affect productivity. Currently, it is not clear whether follow-up work using adaptive laboratory evolution, targeted inhibitor selection, genomic screening, or metabolic engineering to improve substrate utilisation, robustness, or contamination resistance exists. These have direct implications for scalability and reproducibility. A substrate-strain combination may perform well in a single-batch experiment but still fails under scale-up if the organism is sensitive to variable feedstock composition, inhibitory compounds, microbial competition, or repeated-use stress.

### Protein quality assessment

Protein quality refers to the extent to which a protein satisfies an organism’s essential amino acid requirements and overall physiological needs [50]. It is typically evaluated using amino acid scoring approaches that compare the essential amino acid profile of a given protein with established requirement patterns. The strength of protein-quality evidence in the reviewed literature is uneven. Most studies report crude protein, true protein, soluble protein, or related biochemical indicators, but only a smaller subset of studies assess stronger quality parameters, such as the amino acid profile, feed response, or clearly defined mycoprotein yield. Of the sixteen studies summarised in Table 1, only two (refs. 33, 52) report amino acid data, and none report a feeding trial. This difference is important because an increase in crude protein alone does not necessarily demonstrate that the final product has the amino acid balance, digestibility, or safety profile required for practical substitution of conventional protein sources such as soybean meal or fishmeal [51].

Aruna et al. [33]. provide one of the clearest examples of protein-quality assessment beyond crude protein alone. As summarised in Table 1, the fermentation of yam peels with *S. cerevisiae* resulted in marked improvements in both crude and true protein and a more favourable amino acid profile, particularly under ammonium sulfate supplementation. This type of evidence is important because it extends beyond compositional increases and indicates an improvement in protein quality, reflecting better alignment with essential amino acid requirements rather than simple nitrogen enrichment. A second strong example is the *Pleurotus ostreatus* mycoprotein study on sugarcane straw and cassava peels by Adetuwo and Naomi [52]. As shown in Table 1, the study revealed a high mycoprotein yield, substantial protein content, and a balanced amino acid profile under optimised conditions. This represents one of the clearest Nigerian-based examples of a product approaching direct comparability with conventional high-protein feed materials, particularly in crude-protein terms.

**Table 1:** Representative Nigerian studies relevant to waste-derived SCP

Substrate(s)	Microorganism(s)	Bioprocess & Conditions	Protein/biomass determination method	Main reported outcome(s)	Application	Refs.
Yam peels	*Saccharomyces cerevisiae* (BY4743)	SSF, 96 h, with/without (NH₄)₂SO₄	Kjeldahl (*N*×6.25) for crude protein; TCA-precipitable N for true protein	Crude protein 6.6→15.5%; true protein 4.4→13.4%; improved amino acid profile	Potential human food/feed ingredient	[33]
Yam peels	*Aspergillus niger*	Submerged, 7 days	OD₆₅₀ turbidimetry (growth only; no product-protein assay reported)	Biomass OD_650_ 0.40 (yam peel) vs. 0.23 (YEPD). Growth proxy, not a protein value	Low-cost SCP substrate concept	[34]
*Anogeissus leiocarpus* (African birch) sawdust	*A. niger*; *Trichoderma harzianum*	SSF, pH 4–7, 28–40 °C, 6–14 days	Colorimetric soluble-protein assay (Lowry/Bradford-type)	Total soluble protein 157–365 mg/L (*A. niger*), 175–885 mg/L (*T. harzianum*) [≈ 0.157–0.885 g/L]	Industrial bioprotein/enzymes	[41]
*Terminalia superba* (afara) sawdust	*T. harzianum*	SSF, pH 4–7, 28–40 °C, 6–14 days	Colorimetric soluble-protein assay; gravimetric (dry-weight) biomass	Bioprotein 149–346 mg/L [≈ 0.149–0.346 g/L]; biomass 0.08–0.91 g (dry weight; different basis, not a concentration)	Fungal biomass for industry	[53]
Palm kernel meal, groundnut shell, rice bran, wheat bran, corn bran, corn cob, cassava peel	*Pleurotus sajor-caju* (wild-type and UV-mutant strains)	SSF, 7–14 days	Gravimetric (dry-weight) biomass	Biomass yield, g/L (dry weight, at 7→ 14 days): PKM 10.50→17.20; groundnut shell 7.20→13.40; rice bran 6.20→10.40; cassava peel 0.02→0.05	Livestock feed “mycomeat”	[36]
Agricultural substrates as above	*Pleurotus sajor-caju* (wild-type and UV-mutant strains — see note below)	SSF, 7–14 days; UV-exposure study	Gravimetric biomass; mineral analysis (ashing)	Biomass production and mineral composition compared across substrates; PKM highest, cassava peel among poorest	Feed/mycomeat quality	[48]
Sugarcane straw + cassava peels	*Pleurotus ostreatus*	Optimised SSF (RSM): 72:28 mix, 23 °C, pH 6.2, 68% moisture	Gravimetric (yield); Kjeldahl (protein); amino acid profiling	Mycoprotein yield 47.2%; protein 65.1% (w/w); balanced amino acid profile	Human food mycoprotein	[52]
Banana, pineapple, orange, watermelon, cucumber wastes	*Aspergillus niger*	Submerged; nitrogen supplementation	Gravimetric (dry-weight biomass); Kjeldahl (broth protein)	Banana waste highest of those tested: biomass 2.29 ± 0.02 g/L, protein 0.57 ± 0.01 g/L; with NH₄NO₃ supplementation, 3.20 ± 0.02 g/L biomass, 0.79 ± 0.04 g/L protein	SCP for feed/food; enzyme coproduction	[31]
Pineapple and watermelon peels	*Saccharomyces cerevisiae*	Submerged; 16 days; nutrient supplementation	Kjeldahl (SCP %; broth crude protein)	SCP up to 9.8% (pineapple) vs. 2.42% (watermelon); broth crude protein up to 20 g/L with supplementation	Protein ingredient for foods/feeds	[32]
Cassava peels	*Rhizopus oligosporus*; *Saccharomyces cerevisiae*	SSF by R. oligosporus; yeast grown on extract	Soluble protein: assay not stated in source; growth: OD₆₀₀ turbidimetry	Soluble protein reported as increased (4 g/L); yeast growth OD_600_ 0.465 ± 0.005 (fermented extract) vs. 0.113 ± 0.001 (unfermented); growth proxy, not a protein value	Yeast, α-amylase, feed ingredient	[35]
Cassava peels	Mixed yeast isolates from palm wine and grass (“YLEC”)	Semisolid fermentation, 7 days	Colorimetric (soluble protein); Kjeldahl (total protein)	Soluble protein 267.9 mg/g; total protein 11.3%; cyanide reduced to 15.5 mg/kg	Partial maize replacement in broiler feed	[54]
Maize cob	*Rhizopus oligosporus*	SSF, 72 h, pH 6, phosphate buffer	Colorimetric assays (soluble protein, sugars)	At 10% inoculum: glucose 1.15 ± 0.21 mg/g, reducing sugars 45.7 ± 0.6 mg/g, soluble protein 12.9 ± 0.3 mg/g	Poultry feed; safe performance	[40]
Maize offal	*Rhizopus oligosporus*	SSF, pH 3–9, 5 days	Colorimetric soluble-protein assay	Soluble protein increased (421.73 ± 3.00 mg/g at pH 3 vs. 203.87 ± 13.30 mg/g control); glucose and amylase activity also increased	Livestock feed fortification	[39]
Ekpoma rice husk	*S. cerevisiae* (Baker’s yeast); unidentified yeast isolate (palm wine yeast); *Rhizopus oligosporus*	Co-fermentation, 72 h, 30 °C, pH 6.0	Colorimetric soluble-protein assay	Soluble protein 4.7→10.5 mg/g in single-culture fermentations; co-fermentation gave a 183% increase over raw husk	Value-added livestock feed	[43]
Rice bran	*Aspergillus niger* (USM F4)	SSF, 14 days, 25/35/45°C	AOAC proximate methods (Kjeldahl-based crude protein; crude fibre)	Optimal at 35 °C, day 10: crude protein highest, crude fibre lowest (presented in percentage)	Feed ingredient improvement	[47]
Melon, groundnut, walnut shells	*Bacillus subtilis*	Submerged; nitrogen source optimisation; pH 7	Turbidimetric/gravimetric biomass (confirm against source)	Groundnut shell gave highest cell biomass; maxima 0.1888 mg/L (72 h), 0.4580 mg/L (+ yeast extract), 0.3916 mg/L (+ glucose), notably lower than other biomass figures in this table	Bioprotein for feed; waste bioremediation	[42]

Notes on comparability. OD₆₀₀/OD₆₅₀ values are a turbidimetric growth proxy, not a protein quantity, and are reported separately from protein values throughout. Crude protein (Kjeldahl *N*×6.25) includes non-protein nitrogen; true protein excludes it (e.g., via trichloroacetic-acid precipitation); soluble protein is measured by a colorimetric dye-binding assay (e.g., Lowry- or Bradford-type). Concentrations originally reported per litre (mg/L, g/L) are shown on a common g/L scale in brackets. Per-gram soluble-protein values (mg/g) are left in their originally reported units as sources do not consistently state whether “per gram” refers to dry substrate or to an extract, and some related studies in this literature use “%” to denote percentage increase over control rather than composition. UV exposure (refs. 40, 47) was a mutagenesis step used to develop a higher-yielding mutant strain of Pleurotus sajor-caju; wild-type and mutant strains were compared side-by-side via solid-state fermentation directly on the raw substrate (no added liquid, fungus growing on/through the substrate in trays), with biomass harvested at 7 and 14 days

## Challenges and pathways for SCP development in Nigeria

### Current research status and demonstrated feasibility

Research related to SCP in Nigeria has progressed sufficiently to demonstrate clear biological feasibility. However, it remains largely confined to the proof-of-concept and early applied research stages. Numerous studies have shown that locally available microorganisms can grow on cassava peels, yam peels, maize residues, fruit wastes, shell wastes, and other agro-industrial substrates, leading to either direct biomass formation or protein enrichment and broader biochemical upgrading of the substrate [33, 34]. These findings confirm that Nigerian waste streams can support microbial conversion and that the country’s biomass base is scientifically relevant for SCP-related development. A smaller subset of studies moves beyond simple compositional change to provide feed-oriented validation. Fermented cassava-derived materials and fermented maize cobs have been incorporated into broiler diets without adverse effects on growth performance or major serum liver-enzyme indicators, while bacterial protein derived from agro-shell wastes has also been explored as a fish-feed ingredient [40, 54, 55]. However, the current literature still shows limited translational maturity. Majority of studies are laboratory-scale experiments conducted in trays, flasks, jars, or small semisolid systems. Limited evidence exists regarding pilot-scale fermentation, repeated industrially relevant operations, or market-ready product development, as seen elsewhere [56, 57]. The main limitation is therefore not that Nigerian SCP research has failed biologically but rather that most studies were designed to demonstrate feasibility rather than to support commercial translation.

### Constraints to commercial translation and the way forward

**Fig. 3:**
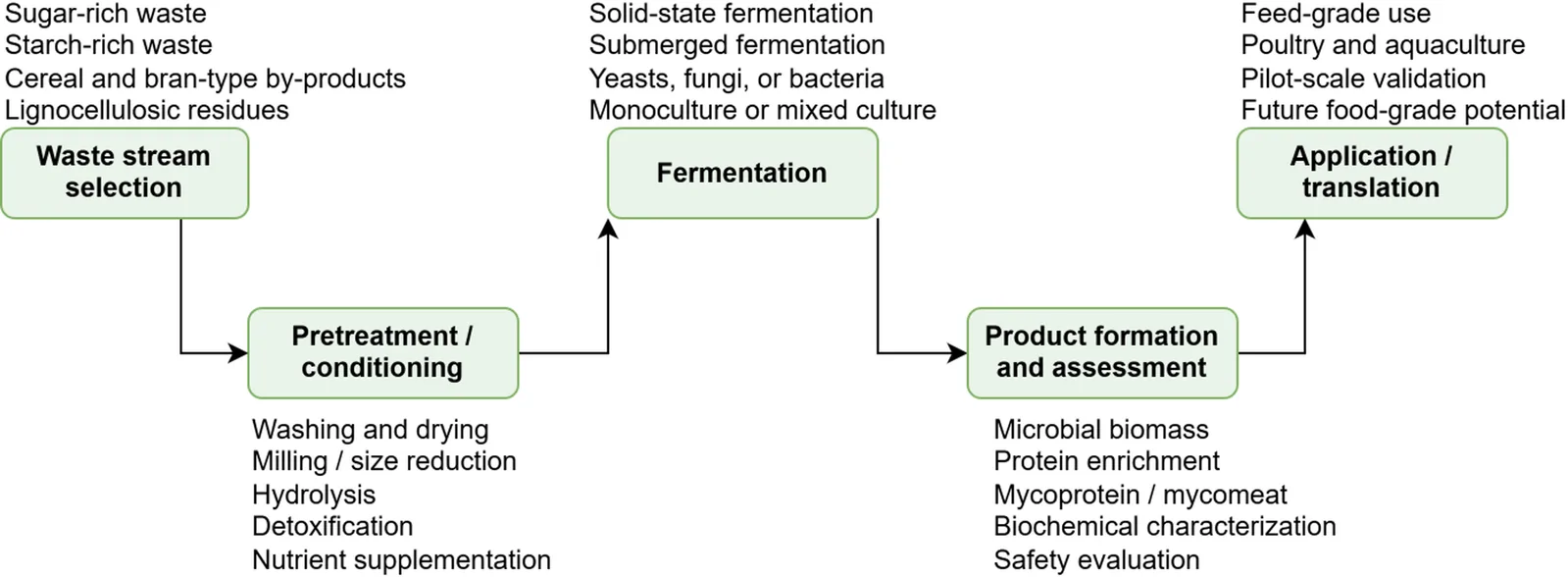
General process flow for waste-derived SCP production in Nigeria

**Fig. 4:**
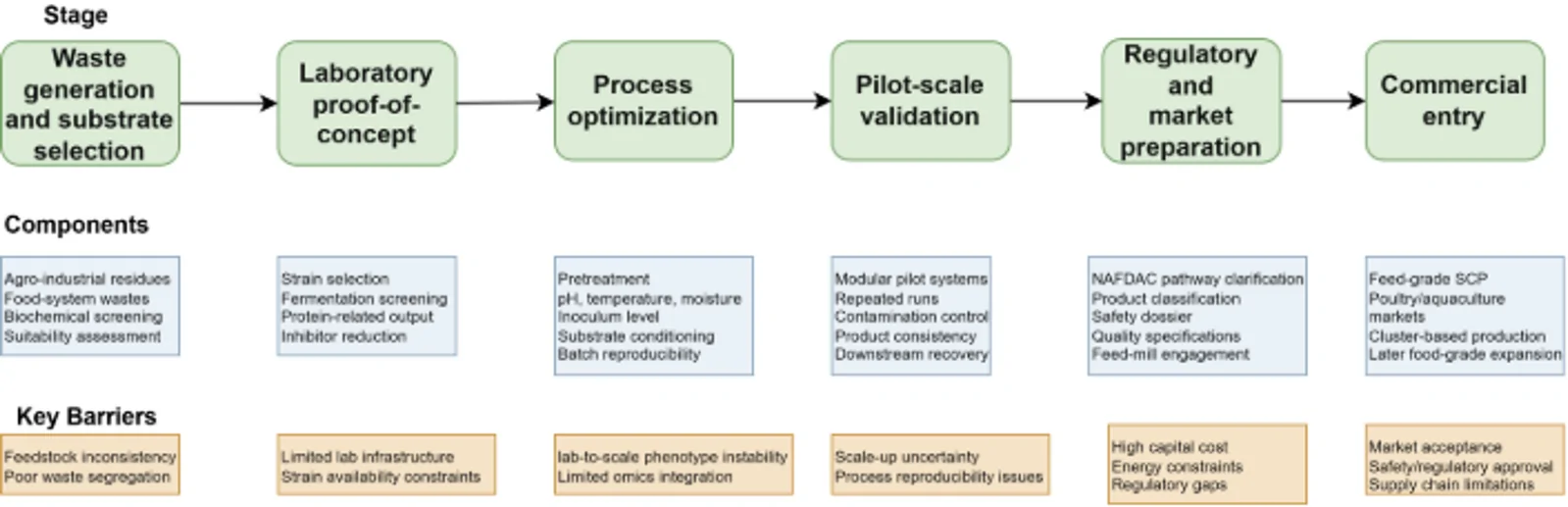
Proposed translational pathway for SCP development in Nigeria

The general process logic linking Nigerian waste streams to SCP-related products is summarised in Fig. 3, while a staged translational pathway is outlined in Fig. 4. The figures show that the main challenge in Nigeria is no only whether local wastes can support microbial conversion but also how to move from laboratory feasibility to reproducible, regulated, and investable production systems. The reviewed literature did not identify any Nigerian pilot-scale, fed-batch, or continuous SCP platform, indicating that the present evidence remains concentrated at the proof-of-concept level rather than industrial translation. The first set of constraints is technical. Most studies are batch experiments carried out in trays, flasks, jars, or small fermenters, which limits confidence in process stability, contamination control, repeated-run reproducibility, and downstream recovery under realistic operating conditions. This is important because many Nigerian substrates are heterogeneous and may vary in moisture, inhibitor burden, and nutrient balance across batches. As a result, the literature is stronger on biological feasibility than on engineering robustness.

A second set of constraints is linked to finances. No Nigeria-specific techno-economic analysis was identified in the reviewed literature, so there is still no reliable local estimate of capital requirements, operating costs, or economically viable production scales. International evidence suggests that SCP economics are highly sensitive to a few major cost drivers, especially feedstock collection and conditioning, pretreatment, energy demand, aeration or agitation, drying, and plant scale. A recent technoeconomic analysis (TEA) of gas-fermentation SCP reported a minimum selling price of approximately US$2,070 per metric ton, but this figure must be interpreted with caution before a comparison with Nigerian conditions is drawn [58]. The underlying process in that study involved the use of hydrogen-oxidising bacteria cultivated on gaseous substrates such as CO₂ and H₂, which require specialised bioreactor infrastructure, precise gas handling, and high capital investment that is similar to the solid-state or submerged fermentation systems on agro-residues that dominate the Nigerian literature. Hence, the US$2,070 benchmark should be read only as an indication of the order of magnitude involved in industrial SCP production rather than as a transferable cost estimate. Nigerian conditions would alter the economic picture in both directions. On the cost-reducing side, locally generated agro-industrial wastes such as cassava peels, yam peels, and maize offal are available at near-zero or very low feedstock cost, and labor costs are substantially lower than in the high-income settings where most international TEAs have been conducted. On the cost-increasing side, unreliable grid electricity often necessitates a diesel-powered backup, increasing energy costs considerably; fermentation and drying equipment is largely import-dependent and subject to foreign exchange volatility; and the logistical infrastructure for consistent waste aggregation and product distribution remains underdeveloped in many production areas. These opposing pressures mean that a Nigerian SCP operation could be cheaper or more expensive than international benchmarks depending on process design, location, scale, and energy strategy, none of which can be resolved without local pilot data. The international literature clearly confirms that without a Nigeria-specific TEA grounded in real pilot-scale data, any claim of cost competitiveness against soybean meal or fishmeal remains speculative.

Another vital constraint to consider is regulation. In Nigeria, the NAFDAC already maintains registration guidance for animal feed made in Nigeria, and related regulatory instruments exist for animal feed and pet food products [59]. In parallel, the Standards Organisation of Nigeria (SON) maintains a dedicated animal needs technical committee [60]. The regulatory landscape differs meaningfully depending on whether the SCP is intended for food or feed-grade or application. For food-grade SCP, no equivalent Nigerian-specific framework currently exists for novel microbial proteins intended for human consumption, and this route would require substantially more safety evidence, including full toxicological assessment and allergenicity profiling, making it a medium-to-long-term prospect rather than an immediate development target. For feed-grade SCP, a more concrete pathway already exists; a registration dossier would typically require product identity, proximate composition, safety and toxicology data, and a description of the manufacturing process. However, uncertainty remains because waste-derived SCP is not yet explicitly classified into a distinct product category, and depending on formulation and intended use, it could plausibly be treated as a feed ingredient, feed additive, or related category ambiguity that affects dossier design, approval timelines, and investor confidence. This regulatory asymmetry further explains why the feed-grade SCP remains the most realistic near-term entry point. Nigeria already has a large feed-protein market, and recent market data indicate approximately 1.05 million metric tons of soy use in animal feed in MY 2023/24, including 775,000 metric tons in poultry and 246,200 metric tons in aquaculture feed. Nigeria also imported approximately 9,258.9 tonnes of fishmeal-related products in 2023, valued at approximately US$20.05 million [12]. These figures do not prove that SCP can immediately substitute for conventional proteins, but they do show that there is a commercially meaningful market for a validated feed-grade microbial protein ingredient.

In line with this, the most realistic pathway forward is a stepwise, feed-oriented translation strategy rather than an immediate attempt at food-grade commercialisation. Priority should be placed on modular pilot plants built around substrates that already show both biological promise and manageable process demands. Suitable candidates include conditioned cassava residues, yam peels, selected fruit wastes, maize offal, and nutrient-rich agro-industrial byproducts. These pilots should be designed not only to produce biomass but also to generate local data now missing from the literature. Key data needs to include substrate logistics, pretreatment cost, energy demand, repeated-run performance, contamination losses, product consistency, and preliminary market-fit information. Scale target is also important. Meaningful pilot operation is generally associated with bioreactor volumes in the range of 100-1,000 L [61]. This range represents the accepted intermediate step between bench-scale experiments and industrial production, and Nigerian efforts should aim to operate within it before any commercial claims can be responsibly made. Product quality targets are equally important. A minimum crude protein content of approximately 40% on a dry weight basis is widely cited as the threshold for feed-grade competitiveness [62]. Nigerian pilot efforts should adopt this as a minimum design target rather than reporting protein enrichment alone. A cost orientation must also be established early. Soybean meal and fishmeal are the two conventional protein sources with which waste-derived SCP could compete. On the basis of recent IMF commodity data, global reference prices stood at approximately US$307 per metric ton for soybean meal and US$1,435 per metric ton for fishmeal [63, 64]. Any Nigerian SCP production cost estimate generated from pilot data should be interpreted against these benchmarks to assess genuine competitiveness. Colocation with cassava processors, rice mills, breweries, or palm oil processing clusters would further improve substrate consistency and reduce transport costs. Early regulatory engagement should run in parallel with pilot development. This means clarifying at an early stage whether the intended product is to be positioned as a feed ingredient or a related category and then generating the identity, safety, composition, and quality-control data required for that route. Moreover, pilot projects should be linked to potential downstream users in poultry and aquaculture feed so that formulation trials and market acceptance are considered from the outset rather than after technical development is complete.

## Conclusion

This review shows that Nigeria already has a meaningful scientific foundation for the development of waste-derived SCP. Across the studies reviewed, a wide range of local waste streams supported microbial growth, protein enrichment, or direct biomass formation, indicating that Nigerian organic residues can serve as useful substrates for SCP-related applications. However, the evidence base still strongly promotes laboratory feasibility rather than commercial readiness. Most studies remain small-scale, report performance using nonuniform metrics, and provide only limited information on protein quality, process robustness, or regulatory preparedness. A major insight from this review is that future progress in Nigeria will depend less on demonstrating that SCP production is possible and more on building systems that are practical, reproducible, and ready for translation. Substrates with better microbial usability and lower pretreatment demands appear more suitable for near-term development, whereas feed-grade SCP stands out as the most realistic entry point because it fits more closely with current market demand and regulatory practicality than immediate food-grade application does.

## Data Availability

No datasets were generated or analysed during the current study.
